# Cardiovascular Autonomic Neuropathy Contributes to Sleep Apnea in Young and Lean Type 1 Diabetes Mellitus Patients

**DOI:** 10.3389/fendo.2014.00119

**Published:** 2014-08-11

**Authors:** Carolina Castro Porto Silva Janovsky, Luiz Clemente de Souza Pereira Rolim, João Roberto de Sá, Dalva Poyares, Sergio Tufik, Ademir Baptista Silva, Sergio Atala Dib

**Affiliations:** ^1^Endocrinology and Diabetes Division, Medicine Department, Universidade Federal de São Paulo, São Paulo, Brazil; ^2^Sleep Medicine Division, Psychobiology Department, Universidade Federal de São Paulo, São Paulo, Brazil; ^3^Clinical Neurology Division, Neurology and Neurosurgery Department, Universidade Federal de São Paulo, São Paulo, Brazil

**Keywords:** diabetic autonomic neuropathy, diabetic complications, heart function tests, obstructive sleep apnea, polysomnography, type 1 diabetes mellitus

## Abstract

Knowledge about association between sleep apnea and cardiovascular autonomic neuropathy (CAN) in type 1 diabetes mellitus (T1DM) might give some insight into the pathogenesis of this condition in these patients. In obese patients, excessive central adiposity, including a large neck circumference, can contribute to obstructive sleep apnea (OSA). Its presence in non-obese patients, however, indicates that it could be correlated with autonomic neuropathy. The aim of this study was to compare the prevalence of OSA in young and lean T1DM patients with and without CAN. We studied 20 adult, non-obese, T1DM patients who were divided into two groups according to the results of the cardiovascular autonomic reflex tests (CARTs). These two groups (9 with CAN and 11 without CAN) were compared to a control group of 22 healthy individuals, who were matched by age and BMI. A polysomnography was performed and sleep was analyzed. The CAN+ group had a significantly higher prevalence of sleep apnea compared to the other groups (67% CAN+; 23% CAN−; 4.5% controls: CAN+ vs. Control; *p* = 0.006 and CAN+ vs. CAN−; *p* = 0.02). The CAN− group had higher sleep efficiency compared to the CAN+ group, demonstrating impaired sleep architecture in diabetics with this chronic complication. In conclusion, OSA may be related to the presence of CAN in young and lean T1DM patients. It could contribute to worse the prognosis and reducing the quality of life of these patients without specific treatment of these conditions.

## Introduction

Obstructive sleep apnea (OSA) in diabetic patients has been frequently explained by obesity associated with type 2 diabetes (T2DM) (1). However, little is known about the presence of OSA in young, non-obese individuals with type-1 diabetes (T1DM).

During sleep, respiratory function is partially controlled by the autonomic nervous system (ANS). When this system is impaired, as in diabetic autonomic neuropathy, the airway may be less functional and breathing control more variable, resulting in OSA (2, 3). Hence, dysautonomy may favor the occurrence of OSA in diabetic patients regardless of age and weight.

Exploring the relationship between sleep apnea and autonomic dysfunction is an important topic of interest because it impacts both patient care and quality of life (4). Therefore, the knowledge of the prevalence of OSA in T1DM and its relationship with CAN could have a high clinical benefit.

The aim of this study was to compare the prevalence of OSA in young, lean T1DM, with and without CAN.

## Research Design and Methods

We recruited 20 adult T1DM patients with a >5-year diagnosis with intensive insulin therapy from the Diabetes Center of the Federal University of São Paulo. The control group (C group) consisted of 22 healthy individuals, medical students from the Federal University of Sao Paulo; all were young and none were obese (BMI <25 kg/m^2^). The subjects were enrolled after they signed informed consent forms. The protocol was approved by the local ethical committee and was in accordance with the Helsinki Declaration. All enrolled individuals completed the study.

The most recent clinical and laboratory data were taken from the patients’ Diabetes Center file. Patients with clinical nephropathy or non-treated thyroid disease were excluded from the analysis.

Autonomic neuropathy was assessed by the following standard protocol of cardiovascular autonomic reflex tests (CARTs) (5) with continuous monitoring of electrocardiography: (1) heart rate variations with deep breathing; (2) heart rate response to the Valsalva maneuver; (3) heart rate changes when standing from the lying position; and (4) postural fall in systolic BP (6). A physician specialized in diabetic neuropathy performed the exam in these controls individuals and diabetic patients at the Diabetes Center early in the morning. The controls and the patients have fasted for the 8 h prior to testing and were not hypoglycemic. The presence of CAN was defined by at least two positive tests (out of four).

Additionally, all subjects were sent to the sleep lab (UNIFESP Sleep Institute) at 8 p.m. to perform the polysomnography exam. There, they were first tested with the Epworth Somnolence Score (ESS), a score that subjectively measures the excessive daytime sleepiness by asking questions about the probability of sleeping in some situations, i.e., watching TV, driving, or waiting at the doctor’s office. The ESS was considered positive for scores as high as nine points (7).

Afterwards, the polysomnography (ALICE – Phillips) was performed according to the recommendations of the American Sleep Disorders Association (7). The exam was performed at night, from 10 p.m. to 6 a.m., and the patients had to avoid caffeine and alcohol 48 h before the exam. The medication was maintained as usual, except for psychotropic drugs, which were withdrawn 1 week before the exam. Sleep data were analyzed with consideration for the sleep latency, duration, stages and efficiency, periodic movements of the limbs, snoring, microarousals, obstructive apnea/hypopnea index (OSAHI), and saturation. Continuous electrocardiogram and electroencephalogram were used to monitor the subjects, and cameras with infrared light were used to monitor the patient.

Obstructive sleep apnea was defined by an OSAHI that was higher than 5/h in the polysomnography. We defined hypopnea as an airflow reduction of 30–50% and apnea as >50%. The microarousal index was considered positive when there were more than 14 events/h (7). The statistical analysis was performed by Pearson chi-square for categorical variables and *t*-tests for continuous variables. A univariate regression test was performed to confirm the homogeneity of the groups. A *p*-value <0.05 was considered significant (Statistic 7.0 Stat Soft Inc.; Tulsa, OK, USA).

## Results

We evaluated 22 non-obese young adults without diabetes (Control group = C) and 20 patients with type 1 diabetes (T1DM) who were also young and non-obese. We divided the T1DM patients into two groups, positive [*n* = 9 (CAN+)] and negative [*n* = 11; (CAN−)], according to the CAN protocol results (6, 8, 9).

Comparing the diabetic group with the control group, the prevalence of excessive daytime somnolence, measured by the EES, and sleep apnea was significantly higher in the diabetic group (*p* = 0.006 and *p* = 0.002, respectively).

The comparisons of clinical, glycemic control, and polysomnography variables between controls, CAN (+) and CAN (−) T1DM patients are shown in Table 1.

**Table 1:** **Clinical, metabolic characteristics, and polysomnography parameters in controls and T1DM patients with (CAN+) and without (CAN−) cardiovascular autonomic neuropathy**.

	Control group	T1DM CAN (–)	T1DM CAN (+)	*p*
Age (years)	23.2 ± 3.9	27.0 ± 8.2	32.7 ± 6.8	NS
BMI (kg/m^2^)	21.8 ± 2.14	22.1 ± 1.8	24.5 ± 2.5	NS
HbA1c (%)	N/A	7.5 ± 1.7	9 ± 2.3	NS
Time from diabetes diagnosis (years)	N/A	16.7 ± 7.8	14.8 ± 5.3	NS
**ESS >9 (% of patients)**	**18.8**	**45.4**	**55.5**	**Control vs. T1DM, 0.002; CAN(−) vs. CAN(+), 0.03**
Total sleep time (min)	415.9 ± 38.8	379.0 ± 50.8	359.1 ± 51.1	NS
**Sleep Efficiency (%)**	**85.0 ± 9.4**	**88.4 ± 5.5**	**80.2 ± 10.7**	**Control vs. T1DM, 0.002; CAN(−) vs. CAN(+), 0.04**
**Microarousals (events/h)**	**7.0 ± 5.0**	**7.7 ± 4.1**	**12.4 ± 5.1**	**Control vs. T1DM, 0.0003; CAN(−) vs. CAN(+), 0.03**
REM (%)	15.9 ± 5.4	17.3 ± 2.8	17.5 ± 8.0	NS
SWS^a^ (%)	20.3 ± 7.2	22.6 ± 6.8	18.9 ± 9.6	NS
**OSAHI^b^ (events/h)**	**2.0 ± 2.3**	**3.2 ± 1.9**	**6.4 ± 4.6**	**Control vs. T1DM, NS; CAN(−) vs. CAN(+), 0.04**
**OSAHI^b^ (% of patients)**	**4.5**	**23.0**	**66.6**	**Control vs. T1DM, 0.006; CAN(−) vs. CAN(+), 0.01**
Desaturation (%)	4.2 ± 0.9	2.9 ± 2.3	4.3 ± 2.5	NS

*Values are expressed as the mean and standard deviation (SD). Bold font indicates statistically significant results*.

*REM, rapid-eye movement*.

*^a^SWS, slow wave sleep (stages 3 and 4)*.

*^b^OSAHI, obstructive sleep apnea/hypopnea index*.

*N/A, not available; NS, non-significant*.

According to a univariate regression test, there was no difference in the age of the studied groups.

The mean HbA1c level and the treatment schedule of these patients were similar to the general population of T1DM patients in São Paulo Federal University Diabetes Center.

## Discussion

This is the first study reporting that young, lean, and long-duration T1DM patients have more OSA than healthy age- and BMI-matched individuals.

Additionally, in lean and young T1DM patients, those with CAN have a higher prevalence of OSA, excessive daytime somnolence, and worse sleep architecture.

Because OSA is an uncommon finding in young and lean individuals, this high prevalence in type 1 diabetes patients, with this profile, suggests that CAN may play an important role in its physiopathology.

Cardiovascular autonomic neuropathy may be concomitant with the damage of vagal reflexes, affecting the upper airway integrity and leading, at last, to sleep apnea (10).

During normal sleep, in contrast to during wakefulness, the parasympathetic ANS controls slow wave sleep (non-REM), reducing sympathetic nerve traffic, heart rate, and blood pressure (10). On the other hand, the sympathetic ANS is responsible for the rapid-eye movement (REM) phase, resulting in intermittent and brief surges in the blood pressure and heart rate. These organized responses to normal sleep are completely disrupted in patients with OSA (2, 3, 10). OSA and hypopneas commonly result in arousals, hypoxemia, and hypercapnia, which are associated with an increase in sympathetic activity and a decrease in the parasympathetic activity, leading not only to higher cardiovascular risk but also to higher oxidative stress and potentially CAN impairment (10–18).

Additionally, because OSA can aggravate and amplify oxidative stress and, ultimately, CAN, the former could contribute to the latter, resulting in a vicious circle. It is important to keep in mind that prospective studies with a larger group of type 1 diabetic patients are needed to determine the mutual relationship between OSA and CAN. However, we can at least speculate that the positive impact of CPAP treatment on oxidative stress and CAN could have significant implications in the prognosis and quality of life of these patients.

Furthermore, we can hypothesize that because diabetes and OSA are associated with increased cardiovascular morbidity and mortality, the presence of both conditions results in additive or even synergistic health risks (19, 20).

To evaluate the prevalence of sleep apnea, we performed a polysomnography, the gold standard test for diagnosis (7). We know that the first night effect might impact our results. However, all patients followed the same protocol and so the results could be comparable.

Recently, studies have shown that the OSA *per se* may impact the levels of HbA1c and the quality of life (4, 21, 22). This fact makes the early diagnosis and treatment of this sleep disturbance even more important for avoiding further chronic diabetes complications in these patients.

However, long-term follow-up studies, as we discussed previously, are needed to determine whether OSA increases the predictive power of autonomic neuropathy for cardiovascular mortality. Spectral analysis during the sleep could help us determine whether these patients are more likely to have sympathetic or parasympathetic damage during sleep.

In conclusion, OSA may be related to the presence of CAN in young and lean T1DM patients.

## Author Contributions

Carolina Castro Porto Silva Janovsky collected the data and wrote the manuscript. Luiz Clemente de Souza Pereira Rolim collected the data, contributed to the discussion, and reviewed the manuscript. João Roberto de Sá contributed to the discussion and reviewed the manuscript. Dalva Poyares collected data and contributed to the statistical analysis. Sergio Tufik collected data. Ademir Baptista Silva collected data, contributed to the discussion, and reviewed manuscript. Sergio Atala Dib wrote and reviewed the manuscript.

## Conflict of Interest Statement

The authors declare that the research was conducted in the absence of any commercial or financial relationships that could be construed as a potential conflict of interest.

## References

[1] Foster GD Sanders MH Millman R Zammit G Borradaile KE Newman AB Obstructive sleep apnea among obese patients with type 2 diabetes. Diabetes Care (2009) 32:1017–9 10.2337/dc08-1776 19279303 PMC2681024

[2] Guilleminault C Briskin JG Greenfield MS Silvestri R The impact of autonomic nervous system dysfunction on breathing during sleep. Sleep (1981) 4:263–78 7302457 10.1093/sleep/4.3.263

[3] Catterall JR Calverley PMA Ewing DJ Shapiro CM Clarke BF Douglas NJ Breathing, sleep, and diabetic autonomic neuropathy. Diabetes (1984) 33:1025–7 10.2337/diabetes.33.11.1025 6500185

[4] Perfect MM Patel PG Scott RE Wheeler MD Patel C Griffin K Sleep, glucose, and daytime functioning in youth with type 1 diabetes. Sleep (2012) 35(1):81–8 10.5665/sleep.1590 22215921 PMC3242691

[5] Rolim LC de Souza JST Dib SA Tests for early diagnosis of cardiovascular autonomic neuropathy: critical analysis and relevance. Front Endocrinol (2013) 4:173 10.3389/fendo.2013.00173 PMC3822331 24273533

[6] Ewing DJ Clark BF Diagnosis and management of diabetic autonomic neuropathy. BMJ (1982) 285:916–8 10.1136/bmj.285.6346.916 6811067 PMC1500018

[7] American Sleep Disorders Standards of Practice Committee. Practice parameters for the indications for polysomnography and related procedures. Sleep (1997) 20:406–22 9302725

[8] Freeman R Autonomic peripheral neuropathy. Lancet (2005) 365:1259–70 10.1016/S0140-6736(05)74815-7 15811460

[9] Boulton AJ Vinik AI Arezzo JC Bril V Feldman EL Freeman R Diabetic neuropathies: a statement by the American Diabetes Association. Diabetes Care (2005) 28:956–62 10.2337/diacare.28.4.956 15793206

[10] Kryger MH Roth T Dement WC Principles and Practice of Sleep Medicine. 4th ed Philadelphia, PA: Elsevier Saunders (2005).

[11] Neumann C Martinez D Schmid H Nocturnal oxygen desaturation in diabetic patients with severe autonomic neuropathy. Diabetes Res Clin Pract (1995) 28:97–102 7587925 10.1016/0168-8227(95)01053-g

[12] Ficker JH Dertinger SH Siegfried W König HJ Pentz M Sailer D Obstructive sleep apnoea and diabetes mellitus: the role of cardiovascular autonomic neuropathy. Eur Respir J (1998) 11:14–9 10.1183/09031936.98.11010014 9543264

[13] Tantucci C Scionti L Bottini P Dottorini ML Puxeddu E Casucci G Influence of autonomic neuropathy of different severity on the hypercapnic drive to breathing in diabetic patients. Chest (1997) 112:145–53 10.1378/chest.112.1.145 9228370

[14] Tahrani AA Ali A Raymond NT Begum S Dubb K Mughal S Obstructive sleep apnea and diabetic neuropathy. Am J Respir Crit Care Med (2012) 186(5):434–41 10.1164/rccm.201112-2135OC 22723291 PMC3443800

[15] Bottini P Dottorini ML Cordoni MC Casucci G Tantucci C Sleep-disordered breathing in nonobese diabetic subjects with autonomic neuropathy. Eur Respir J (2003) 22:654–60 10.1183/09031936.03.00070402 14582920

[16] Keller T Hader C De Zeeuw J Rasche K Obstructive sleep apnea syndrome: the effect of diabetes and autonomic neuropathy. J Physiol Pharmacol (2007) 58(Suppl 5(Pt 1)):313–8 18204141

[17] Mondini S Guilleminault C Abnormal breathing patterns during sleep in diabetes. Ann Neurol (1985) 17:391–5 10.1002/ana.410170415 4004160

[18] Rees PJ Prior JG Cochrane GM Clark TJ Sleep apnoea in diabetic patients with autonomic neuropathy. J R Soc Med (1981) 74:192–5 7205856 10.1177/014107688107400306 PMC1438271

[19] Borel AL Benhamou PY Baguet JP Halimi S Levy P Mallionand JM High prevalence of obstructive sleep apnoea syndrome in a type 1 diabetic adult population: a pilot study. Diabet Med (2010) 27:1328–9 10.1111/j.1464-5491.2010.03096.x 20950392

[20] Diabetes Control and Complications Trial/Epidemiology of Diabetes Interventions and Complications (DCCT/EDIC) Research Group Nathan DM Zinman B Cleary PA Backlund JY Genuth S Modern-day clinical course of type 1 diabetes mellitus after 30 years’ duration: the diabetes control and complications trial/epidemiology of diabetes interventions and complications and Pittsburgh epidemiology of diabetes complications experience (1983-2005). Arch Intern Med (2009) 169(14):1307–16 10.1001/archinternmed.2009.193 19636033 PMC2866072

[21] Tamura A Kawano Y Watanabe T Kadota J Obstructive sleep apnea increases hemoglobin A1c levels regardless of glucose tolerance status. Sleep Med (2012) 13:1050–5 10.1016/j.sleep.2012.04.007 22763014

[22] Villa MP Multari G Montesano M Pagani J Cervoni M Midulla F Sleep apnoea in children with diabetes mellitus: effect of glycaemic control. Diabetologia (2000) 43:696–702 10.1007/s001250051365 10907113

